# Perception of professional ethics by Iranian occupational therapists working with children

**Published:** 2015-05-23

**Authors:** Minoo Kalantari, Mohammad Kamali, Soodabeh Joolaee, Mehdi Rassafiani, Narges Shafarodi

**Affiliations:** 1 PhD Candidate in Occupational Therapy, Department of Occupational Therapy, School of Rehabilitation Sciences, Iran University of Medical Sciences, Tehran, Iran;; 2 Associate Professor, Department of Rehabilitation Management, School of Rehabilitation Sciences, Iran University of Medical Sciences, Tehran, Iran;; 3 Associate Professor, Center for Nursing Care Research, School of Nursing & Midwifery, Iran University of Medical Sciences, and Iranian Academy of Medical Sciences, Tehran, Iran;; 4 Associate Professor, Pediatric Neuro-rehabilitation Research Center, Department of Occupational Therapy, University of Social Welfare and Rehabilitation Sciences, Tehran, Iran;; 5 Assistant Professor, School of Rehabilitation Sciences, Department of Occupational Therapy, Iran University of Medical Sciences, Tehran, Iran.

**Keywords:** *occupational therapy*, *professional ethics*, *children*

## Abstract

Ethics are related to the structure and culture of the society. In addition to specialized ethics for every profession, individuals also hold their own personal beliefs and values. This study aimed to investigate Iranian occupational therapists’ perception of ethical practice when working with children. For this purpose, qualitative content analysis was used and semi-structured interviews were conducted with ten occupational therapists in their convenient place and time. Each interview was transcribed and double-checked by the research team. Units of meaning were extracted from each transcription and then coded and categorized accordingly.

The main categories of ethical practice when working with children included personal attributes, responsibility toward clients, and professional responsibility. Personal attributes included four subcategories: veracity, altruism, empathy, and competence. Responsibility toward clients consisted of six subcategories: equality, autonomy, respect for clients, confidentiality, beneficence, and non-maleficence. Professional responsibility included three subcategories: fidelity, development of professional knowledge, and promotion and growth of the profession. Findings of this study indicated that in Iran, occupational therapists’ perception of autonomy, beneficence, non-maleficence, fidelity and competence is different from Western countries, which may be due to a lower knowledge of ethics and other factors such as culture. The results of this study may be used to develop ethical codes for Iranian occupational therapists both during training and on the job.

## Introduction

Competence and sufficiency in a profession is a combination of knowledge, skill and conduct. Knowledge and skill are formed through professional education, experience and continued learning, but these factors are not the sole requirements for creating a capable and reliable therapist. A therapist should behave in a way that would promote and maintain the welfare of the society and protect the reputation of employers and the profession (1).

Rapid scientific and technological advances in health sciences have influenced research related to occupational therapy. Meanwhile, one important concern is the graduates’ ability to think critically and make decisions based on correct values and professional ethics (2). Therefore, occupational therapy associations and boards in different countries have described core values and professional ethics according to their culture and needs (3-6). Values are a set of beliefs to which an individual is committed, and form an important part of any profession. Moreover, actions and attitudes are the reflections of individual values. Attitudes are our tendencies in giving positive or negative responses to an object, individual, concept or situation. All professional actions and interactions, therefore, originate from values and beliefs (3).

Although there are specific professional values in every line of work, individuals also hold their own personal beliefs and values. Ethics are related to the structure and culture of the society (7), and provision of occupational therapy services without a link to the values and needs of the community is detrimental to the clients in the long run (8). In order to develop appropriate ethical codes and professional behavior in the field of occupational therapy in Iran, the elements of ethics and ethical practice must be identified from the points of view of Iranian occupational therapists.

Occupational therapists work with different age groups that may suffer from physical, mental or social disabilities or limitations. Children are very vulnerable due to their age and decision-making capacity (9). The present study aimed to describe Iranian occupational therapists’ perception of professional ethics when working with children.

## Method

This study was conducted using a conventional qualitative content analysis. Ethical issues and implications of ethical practice in pediatric occupational therapy were investigated based on the experiences of Iranian occupational therapists. It should be added that qualitative content analysis is a method for subjective interpretation of written content through a process of systematic classification of codes and determination of themes and patterns (10).

**Setting and Participants**

Participants in this study included ten (5 female and 5 male) Iranian occupational therapists selected by purposeful sampling. Two of the occupational therapists were PhD students, three of them had master’s degrees, and the remaining five had bachelor’s degrees in occupational therapy. All participants had between 6 and 25 years of experience in working with children. In selecting the participants, we tried to consider maximum diversity in gender, work experience, academic degree and workplace. All participants took part in this study voluntarily. One of the participants worked in a hospital, one in a school for special education, two in centers of the State Welfare Organization, four in private clinics, and two in university clinics. Four participants also provided home care services. The study was conducted between 2013 and 2014 in Tehran, Iran.

**Data Collection**

Data were collected through a total of 11 semi-structured interviews with ten participants at their workplaces. Duration of interviews varied between 30 and 60 minutes. Research goals were explained to the participants before the interviews, and their written informed consent was obtained. Participants were encouraged to share their experiences of professional ethics in pediatric occupational therapy, and then exploratory questions were asked for more detailed information. Interviews continued until data saturation, that is, until the data were repeated and no new information emerged. 

**Ethical Considerations**

The present study was approved by the Ethics Committee of Iran University of Medical Sciences. After providing the necessary information, written informed consent was obtained from all participants. Consent was also obtained for recording the interviews. Participants were assured that the data would be kept confidential, and the interviews were coded accordingly. The participants were told that they had the right to withdraw from the study at any time.

**Data Analysis**

Recorded interviews were transcribed and then analyzed using a content analysis approach. Each interview was double-checked by coauthors, and units of meaning were extracted and then coded and categorized. The analysis process was repeated upon addition of each interview and the codes and categories were modified (10). In this study, credibility, dependability and confirmability measures were used in order to determine accuracy. For this purpose, prolonged engagement was attained by dedicating approximately 6 months to performing interviews and obtaining codes. Diversity in participants was another measure that was employed to enrich data, and therefore participants with different academic degrees and job experiences were selected from various settings. The participants were allowed to review their transcribed interviews to help them maintain consistency. During several meetings our research team members modified and revised the extracted codes until reaching a consensus, and for external check, an expert occupational therapist, who was familiar with both qualitative research and working with children, reviewed the results in the context. 

## Results

Three categories were extracted in connection with ethical practice when working with children: personal attributes, responsibility toward clients, and professional responsibility. Each category included some subcategories (12 subcategories in total), which described specific aspects of ethical practice when working with children (see Table 1).

**Table 1:** Categories and subcategories extracted from the participants’ responses

**Categories**	**Subcategories**
Personal Attributes	Veracity Altruism Empathy Competence
Responsibility toward Clients	Equality Respect for Clients Confidentiality Autonomy Non-Maleficence Beneficence
Professional Responsibility	Fidelity Development of Professional Knowledge Promotion and Growth of the Profession

***Personal Attributes***

The subcategories of personal attributes are as follows:

          *1) Veracity*

Our participants believed veracity to be of high importance in providing services. Truthfulness with clients and their family members, providing realistic information, avoiding deception and creating unrealistic expectations were among the ideas mentioned by most participants. One occupational therapist said:

*“I realized that I should be honest if I want to be effective. This is the ethical rule of veracity.”* [Occupational Therapist 6]

Some occupational therapists preferred veracity over their financial interests when expressing the prognosis. One participant said:

*“At times, I have had to explicitly tell the kid’s prognosis to the mother, even though I knew that she might not bring her kid to the clinic again.”* [Occupational Therapist 2]

          *2) Altruism*

The participants believed that it was one of the personal attributes of a good occupational therapist to do their job in any condition and provide appropriate services even when there is no supervision. One occupational therapist said:

*“Sometimes I was not in a good mood, but I provided all services as if someone was supervising me. It was very important for me. I adhere to ethical principles.”* [Occupational Therapist 2]

          *3) Empathy*

The participants pointed out that understanding the family, considering their financial situation and helping them were among the personal attributes of an occupational therapist. One of our participants said:

*“I have tried to develop empathy with them and help them as far as I could, and to give a discount if they had financial problems. I have received nothing for some sessions.”* [Occupational Therapist 5]

          *4) Competence*

The participants believed that occupational therapists should keep their professional knowledge current, as this depicts their competence for providing therapeutic interventions.

*“Sufficient knowledge of therapeutic interventions is very important because the negative consequences of mistakes in interventions may be much more irrecoverable in children than in adults. Occupational therapists should have up-to-date information.”* [Occupational Therapist 10]

Definition of competence, however, seemed to be different for each participant. On the subject of recruiting students in private clinics before their graduation, one occupational therapist said:

*“If supervised, students in their final semester can work because 8* ^th^ *semester students have enough experience to work.”* [Occupational Therapist 9]

***Responsibility toward Clients***

This category mainly focuses on ethical practice by way of equality, respect for clients, confidentiality, autonomy, non-maleficence, and beneficence.

          *1) Equality*

Non-discrimination in provision of services was a fact indicated by most participants, who believed that the cultural, religious and social conditions of families should not lead to any discrimination among the patients. One occupational therapist said:

*“The cultural, religious and social conditions of a family have no effect on admission of patients. [Factors like that] do not affect me. I had a patient coming from Iraq, another one was very religious, another not. I try to carry out my duties.”* [Occupational Therapist 9]

In order to establish social equality, some occupational therapists tried to make use of public facilities for their patients so that financial problems would not prevent them from continuing treatment. One of the participants said:

*“… I have written letters for them to receive money from charities so that they could continue their sessions and not miss any.”* [Occupational Therapist 2]

          *2) Respect for Clients*

The participants believed that observing children’s rights and respecting their culture and religion were important issues that should be considered when providing services. One occupational therapist believed that observance of this ethical rule would attract clients because families also expect respect.

*“The most important part of communication is to respect the patients. Both the patients and their families should feel respected. What attracts patients is observance of these ethical rules because their families understand that they are respected.”* [Occupational Therapist 9]

Another occupational therapist saw inappropriate conditions in clinics as disrespectful to the clients and stated:

*“I cannot work in a humid and dark basement. It is not human. I want to provide human services, so the conditions should be suitable for humans.”* [Occupational Therapist 6]

          *3) Confidentiality*

According to the participants, professional ethics require that occupational therapists keep patients’ information confidential and respect their privacy. One participant believed:

*“Rules of professional ethics should be observed and the patient’s information should be kept confidential. Therapists should not take off the child’s clothes for examination in the presence of others. The child or the family, especially those with strong religious beliefs, may not approve of that.”* [Occupational Therapist 6]

          *4) Autonomy*

Our participants believed that giving the family the right to decide about their responsibilities is among the rules that should be preserved for the patients.

*“Before discharging a patient, I explain to the family that the sessions are enough in my opinion and the kid needs no further sessions, and tell them that further treatment is up to them.”* [Occupational Therapist 1]

Moreover, most participants stated that in working with children, their consent is a must and they should not be forced to cooperate. One occupational therapist said:

*“I try to establish rapport with the kid for two or three sessions. They should accept me and adapt to the environment, so that I can work with them.”* [Occupational Therapist 3]

The participants believed that families should receive the necessary information about the techniques and therapeutic procedures. Some occupational therapists emphasized the education of families and their presence during the sessions.

*“I work for 30 minutes, and train the mother for 15, and the family attends the treatment session.”* [Occupational Therapist 7]

Another occupational therapist said:

*“I certainly involve the family in treatment. In my opinion, the role of families in the treatment process is close to 80%. But I do not ask their opinion in setting the goals because they may not have realistic expectations.”* [Occupational Therapist 9]

          *5) Non-Maleficence*

The participants stated that they believed it was their duty to avoid behavior that may have potentially harmful consequences. One participant said:

*“Occupational therapists should not practice excessive pressure, as aggression may cause the patient to suffer…. I can only ask them in all seriousness if they did what I said, and how they exercised.”* [Occupational Therapist 6]

In order to protect the patient, one occupational therapist said that if the mental health of the parents is not established, they should not be asked to do the exercises at home.

*“As for procedures that need excessive pressure, if the family is not mentally competent, techniques of violent nature should not be assigned to the mother.”* [Occupational Therapist 6]

“Giving a colleague a hint when facing unethical behavior” was a matter reflected by another occupational therapist:

“*I warn my colleagues when I see unethical behavior, even if it influences my relationship with them.”* [Occupational Therapist 2]

Another occupational therapists showed more flexibility in confronting with unethical behavior:

*“If I somehow feel that they do not misinterpret my words and do not act defensively, and if their characters are known to me, I will give them a hint that it is better not to do so. But at times I have had to remain silent.”* [Occupational Therapist 3]

Another occupational therapist spoke about the steps to follow in this regard:

*“I have warned my colleague several times and I do not care if they feel uneasy. I don’t mind telling the supervisor, and I may even ask the patient to submit a complaint to the ward authorities.”* [Occupational Therapist 10]

          *6) Beneficence*

Our participants all believed that the patient’s interests should be put in priority. One occupational therapist said:

*“The head of the clinic wanted me to increase group therapy sessions to add more cases to my list, but I refused. I said, ‘You are trying to make money, but I am thinking of my job. This kid does not fit in this group at all.’”* [Occupational Therapist 3]

Regarding their responsibility toward patients, one occupational therapist mentioned evidence-based treatment:

*“We cannot apply all techniques to all patients. When it comes to evidence-based treatment, there should be scientific experience on the one hand, and the patient’s demand on the other. The patient should also accept the treatment.”* [Occupational Therapist 6]

The participants believed that they had to inform the family of the child’s condition and ask for their cooperation:

*“Instead of understanding the child’s condition and adapting her expectations to the kid’s ability, the mother regularly shouted at the child. I provided the mother with the necessary information without considering the time and expenses, and helped her to assist the child rather than struggle and make negative remarks.”* [Occupational Therapist 1]

***Professional Responsibility***

This category focuses on professional responsibility and its subcategories, that is, fidelity, development of professional knowledge, and promotion and growth of the profession.

          *1) Fidelity*

One occupational therapist believed that it is not ethical to interfere with another colleague’s job, and when necessary, we should support them rather than defame them:

*“In general, I do not consider it ethical to interfere in the affairs of any specialist. I never comment on my colleague’s performance… I tell the mother that I can’t comment on my colleague’s work when I have not even met them. I cannot make a judgment because I might choose the same course of action if I was in their shoes.” *[Occupational Therapist 3]

          *2) Development of Professional Knowledge*

The participants believed that occupational therapists should consult various resources to obtain the necessary information about the disease, and should always keep their knowledge updated. One occupational therapist said:

*“Typically, I dedicate one session to assessment. If necessary, I search for information… and try to consult experienced specialists. For instance, I tell them ‘The kid has uncontrolled convulsion, do you think it advisable to do these exercises?’”* [Occupational Therapist 9]

One occupational therapist pointed out that we should develop professional knowledge by sharing our experiences:

*“I try to share what I have learned scientifically. I don’t keep things to myself. If I learn a new technique, I make sure to tell others about it.”* [Occupational Therapist 9]

          *3) Promotion and Growth of the Profession*

As viewed by our participants, it is necessary for the promotion and growth of the profession, to avoid inappropriate advertisement. Thus, any action that casts doubts on the performance of occupational therapists should be prevented so that the position and dignity of the profession is maintained. One occupational therapist said:

        *  1) “If someone is making questionable advertisments about the profession, they should be informed. We should preserve our unity. If someone works individualistically, we will be damaged [as members of a profession].”* [Occupational Therapist 9]

Another occupational therapist said:

*“I believe we should not act when in doubt. It is better not to do that… Why should we insist on doing something that may be criticized?”* [Occupational Therapist 6]

Another point emphasized by the participants in relation to the growth of the profession was advertisement and introduction of the profession. One occupational therapist said:

*“Enough information should be provided. Ads will be helpful for public information. They are about introducing our profession [to the public] and the capabilities we have.”* [Occupational Therapist 10]

## Discussion

Findings of this study indicated that ethical practice in working with children could be divided into three categories: personal attributes, responsibility toward clients, and professional responsibility. The participants in this study believed that personal attributes included veracity, altruism, empathy, and competence. World Federation of Occupational Therapists (WFOT) has assigned a code to personal attributes, suggesting that occupational therapists should comply with principles of personal integrity, loyalty, open-mindedness, and reliability in their profession (11). The subcategories extracted in this study have also been mentioned in the ethical codes of different countries. For example, the American Occupational Therapy Association has addressed truthfulness under the title of “veracity” and defined it as conveying “comprehensive, accurate and objective” information to the clients and promoting their comprehension of such information (5). This is necessary for establishing a good partnership between the therapist and the patient (6), and good practice has been found to decrease the possibility of misunderstanding and help occupational therapists avoid moral distress (12). In core values and attitudes of occupational therapy practice, Kanny and Kyler state that the values and attitudes in occupational therapy have been organized around seven main concepts: altruism, equality, freedom, justice, dignity, truth, and prudence (2). Pleoquin identifies empathy as the origin and base of ethics. She believes that empathy is an ethical tendency, and ethical practice is possible through empathy (13). The capacity to empathize with another individuals is the art of practice in occupational therapy (14). In the Code of Ethics and Professional Conduct of the British Occupational Therapy Association (3) and in the Ethical Codes of the Australian Association of Occupational Therapists, competence has been proposed as a professional standard (15). Many mistakes in clinical practice are due to lack of skill, and this explains why competence is included in ethical codes of occupational therapy associations (16). Nevertheless, further studies need to be conducted on the concept of competence among Iranian occupational therapists. Although it is illegal to work before graduation due to lack of sufficient acquaintance with ethical codes and rules, some Iranian occupational therapists recruit senior students in clinics.

Another finding of this study was responsibility toward clients. The participants believed that equality, respect for clients, confidentiality, autonomy, non-maleficence, and beneficence are necessary for ethical practice in working with children. WFOT identifies respect, non-discrimination, consideration of the client’s values and priorities, and confidentiality among the responsibilities of occupational therapists toward all service receivers (11). These concepts have also been included in ethical codes of the United States, Australia and Canada, although the implications are somehow different. For example, in the United Kingdom, Australia and the United States, autonomy means to give the client the right to choose and have active participation in therapeutic decisions and procedures, and the client’s consent is necessary before the treatment process (3, 5, 15). In this study, some participants saw autonomy as seeking the child’s consent and not forcing them to attend treatment sessions, or communicating the treatment plan to the family due to the client’s age. Children are more dependent on others (their parents in particular) compared to adults. The important question here is, who is the actual client, the child or the family? Children can have different views from their parents and should therefore be consulted about the treatment process as far as possible.

In pediatrics, the family-centered approach is more common than the client-centered approach (17). Although being family-centered is a core value in working with children, participation of the family in determining treatment goals and decisions is not fully enforced. Some of our participants suggested that they could not follow the demands of the family because their expectations were not realistic. For most participants, autonomy meant asking about the family’s goals and informing and educating them about the treatment plan. 

The occupational therapists in this study stated that clients do not actively participate in the treatment process and mentioned the inappropriate expectations and low educational level of some families to be the reason. This may be partly due to a lack of moral education in occupational therapists, although some of the participants were PhD students and had passed ethics courses. The study conducted by Pettersen and Svilaas in India showed that culture influenced client-centered treatment, and that Indian occupational therapists tried to apply some elements of client-centered treatment (18). Ludwick and Silva suggested that in some cultures, health decisions were not made by an individual, but by a group, that is, the family or the society. It is therefore necessary to address the values prominent in such cultures in order to promote ethical thinking (19). As for beneficence, some Iranian occupational therapists seem to go to extremes in their commitment to this rule. Although some of our participants believed that the child should be at peace during the sessions, others thought that crying was inevitable and should be ignored to make the best use of the treatments. They stated that some parents preferred that their children be treated with higher intensity. It seems that participants in this study were so involved in beneficence that they overlooked the rule of non-maleficence, which needs further research.

Another category of ethical practice in this study was found to be professional responsibility. The participants believed that professional responsibility included fidelity, development of professional knowledge, and promotion and growth of the profession. With respect to fidelity, Rule 7 of the American Occupational Therapy Code of Ethics states: “Occupational therapy personnel shall treat colleagues and other professionals with respect, fairness, discretion, and integrity” (5). This definition is consistent with the responses of Iranian occupational therapists, although fidelity should not interfere with fulfillment of ethical commitments. If an occupational therapist is in doubt about his/her colleague’s behavior, he/she may submit a complaint to competent authorities (15). Occupational therapists should assume their professional responsibility and deliver safe, ethical and effective treatments and report unethical or incompetent conduct (20). Nevertheless, most participants in this study sidestepped confrontations and did not report unethical conduct to the authorities so as to maintain relationships with their colleagues or avoid isolation. Some participants had passed ethics course and were familiar with ethical rules, but uncertainty was evident in their responses as well. It seems that such behavior stems from the culture rather than unawareness of ethical rules. As a rule, cultural factors influence people’s conduct to a great extent (21), and further studies on this issue are therefore suggested.

The participants believed that development of professional knowledge and promotion and growth of the profession are among our duties in working with children. Development of professional knowledge through continuing and post-professional education programs may provide occupational therapists with the knowledge, skills and attitudes that will help them perceive themselves as competent practitioners (22). Brown (2010) suggested that continuing education may be required to ensure evidence-based treatment and research utilization among pediatric occupational therapists (23). Guidelines on continuing education have been compiled due to the importance of developing professional knowledge (24). For instance in the United Kingdom, occupational therapists are expected to assume responsibility for the education of the public and the promotion of health and welfare in order to decrease the influence of disease and disability (3).

## Conclusion

Based on our findings, the views of Iranian occupational therapists on ethical practice in working with children were similar to those of occupational therapists of Western countries in some aspects. Nevertheless, their understanding of concepts such as autonomy, beneficence, non-maleficence, competence and fidelity were different from those of Western occupational therapists. It can be concluded that various people may develop different interpretations of the same concept. This may be due to lack of education, which should be considered in undergraduate curricula as well as ethical courses and workshops for Iranian occupational therapists. In this regard, other possible factors such as culture should also be taken into account.
